# Daily fluctuating temperatures decrease growth and reproduction rate of a lethal amphibian fungal pathogen in culture

**DOI:** 10.1186/s12898-020-00286-7

**Published:** 2020-04-03

**Authors:** Alexa L. Lindauer, Paul A. Maier, Jamie Voyles

**Affiliations:** 1 Department of Biology, University of Nevada Reno, 1664 N. Virginia Street, Reno, NV 89557 USA; 2 Department of Biology, San Diego State University, 5500 Campanile Drive, San Diego, CA 92182 USA; 3 FamilyTreeDNA, Gene by Gene, 1445 N Loop W, Houston, TX 77008 USA

**Keywords:** *Batrachochytrium dendrobatidis*, Disease ecology, Temperature, Yosemite toad, Sierra Nevada

## Abstract

**Background:**

Emerging infectious diseases (EIDs) are contributing to species die-offs worldwide. We can better understand EIDs by using ecological approaches to study pathogen biology. For example, pathogens are exposed to variable temperatures across daily, seasonal, and annual scales. Exposure to temperature fluctuations may reduce pathogen growth and reproduction, which could affect pathogen virulence, transmission, and environmental persistence with implications for disease. We examined the effect of a variable thermal environment on reproductive life history traits of the fungal pathogen *Batrachochytrium dendrobatidis* (*Bd*). *Bd* causes chytridiomycosis, an emerging infectious disease of amphibians. As a pathogen of ectothermic hosts, *Bd* can be exposed to large temperature fluctuations in nature. To determine the effect of fluctuating temperatures on *Bd* growth and reproduction, we collected temperature data from breeding pools of the Yosemite toad (*Anaxyrus canorus*), a federally threatened species that is susceptible to chytridiomycosis. We cultured *Bd* under a daily fluctuating temperature regime that simulated Yosemite toad breeding pool temperatures and measured *Bd* growth, reproduction, fecundity, and viability.

**Results:**

We observed decreased *Bd* growth and reproduction in a diurnally fluctuating thermal environment as compared to cultures grown at constant temperatures within the optimal *Bd* thermal range. We also found that *Bd* exhibits temperature-induced trade-offs under constant low and constant high temperature conditions.

**Conclusions:**

Our results provide novel insights on variable responses of *Bd* to dynamic thermal conditions and highlight the importance of incorporating realistic temperature fluctuations into investigations of pathogen ecology and EIDs.

## Background

Emerging infectious diseases (EIDs) are increasing in incidence and are responsible for plant and animal population declines in managed and wild systems [20, 29, 68]. To understand the drivers of EIDs, the rapidly developing field of disease ecology integrates traditional approaches of parasite biology into ecological and evolutionary frameworks [16]. One recent focus has been to understand the effects of environmental temperature fluctuations on disease [11, 69]. Differences in magnitude, range, and variability of daily temperature fluctuations have been shown to affect transmission intensity of malaria [40, 45], transmission rates of dengue virus [9, 30], biocontrol of the chagas disease vector [18], and susceptibility of black abalone to withering syndrome [4]. Temperature can profoundly influence disease outcomes due to the thermal sensitivity of host and pathogen traits, including pathogen growth and reproduction [1, 10, 58]. Because pathogen growth and reproduction are tied to virulence [38], understanding the responses of these life history traits to thermal heterogeneity may reveal important patterns in infectious disease.

Temperature can affect the disease ecology of chytridiomycosis [57, 76], a lethal emerging infectious disease of amphibians that is responsible for global amphibian declines [63, 64]. Chytridiomycosis is caused by the pathogenic fungus *Batrachochytrium dendrobatidis* (*Bd*), which has a complex and temperature-sensitive life history [5, 35]. Motile *Bd* zoospores encyst in keratinized amphibian tissues and develop into zoosporangia [5, 48]. Zoosporangia produce the next generation of zoospores and release them into the environment or back onto the amphibian host [6, 35]. This life cycle requires temperatures between approximately 2–27 °C in vitro, with an optimal temperature range between 15 and 25 °C and a drop in reproduction and viability above 27 °C [51, 66, 73, 78]. Because increases in *Bd* loads correlate with the severity of chytridiomycosis [72, 74], exposure to temperatures above the *Bd* thermal maximum that negatively affect *Bd* growth and reproduction may decrease infection intensities and slow disease progression [23, 26, 62].

To date, temperature studies have predominantly focused on *Bd* responses at constant temperatures (e.g. [51, 78]). However, amphibian hosts live in microhabitats with remarkable thermal heterogeneity across daily, seasonal, and annual cycles (e.g. [43, 75]). Constant temperature studies have provided critical insights into *Bd* biology but have not discerned how realistic fluctuating thermal environments may influence *Bd* growth and reproduction. Recent work by Raffel et al. [55] and Greenspan et al. [23] suggest that fluctuating thermal conditions can have profound effects on *Bd* growth in vitro and on chytridiomycosis development in vivo. These studies add to evidence in other disease systems that constant temperature experiments may not be generalizable to disease dynamics in wild populations because thermal fluctuations can have disproportionate biological consequences on pathogen traits [9, 30, 40, 45]. In addition, thermal heterogeneity may influence the persistence of free-living *Bd* in water bodies used by amphibian hosts. While the mechanisms or duration of *Bd* persistence in natural environments remain unclear [7, 41], models suggest that extended environmental persistence of *Bd* outside amphibian hosts is likely to increase local extinction risk [39]. Understanding how dynamic thermal regimes affect *Bd* outside of hosts may be an important conservation tool to target where *Bd* is (and is not) on a landscape [21].

**Fig. 1:**
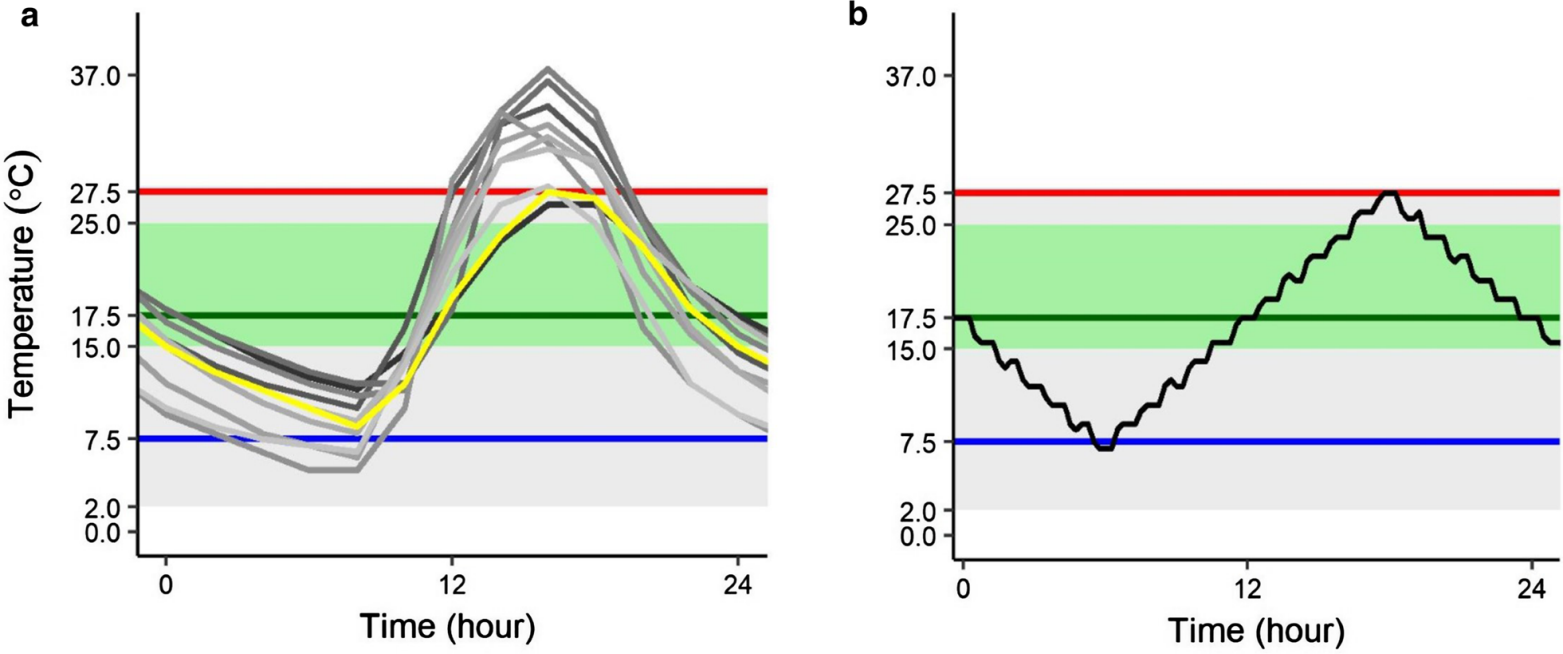
Observed and experimental diurnal temperature fluctuations. **a** Water temperature over a 24-h period of 10 different breeding pools containing Yosemite toad tadpoles (grey lines; yellow line represents pool fluctuating within 27.5 and 7.5 °C). **b** Incubator temperature profiles over a 24-h period. Fluctuating temperature = black; constant temperature at daily thermal maximum (27.5 °C) = red; constant temperature at daily thermal minimum (7.5 °C) = blue; constant temperature at daily thermal mean (17.5 °C) = green. *Bd* thermal optimum (green shaded band) and *Bd* thermal tolerance (grey shaded band) shown for reference

In this study, we examined responses of *Bd* in culture to biologically realistic temperature fluctuations that simulate the thermal conditions of water bodies used by the Yosemite toad (*Anaxyrus* [*Bufo*] *canorus*). The Yosemite toad is a federally threatened California endemic that is highly susceptible to lethal chytridiomycosis in controlled exposure experiments [32]. While *Bd* infection has been detected in all life stages of wild Yosemite toads [19]; C. Dodge unpublished data), the role of *Bd* in the decline of this species is not well understood. Yosemite toads breed and develop in shallow pools in high elevation meadows in the Sierra Nevada Mountains of California, USA that undergo large daily temperature fluctuations compared to the *Bd* thermal range (Fig. 1a; [13, 28, 43]). However, it is unclear how these temperature fluctuations affect *Bd* growth and reproduction and in turn, the disease ecology of this system.

To better understand *Bd* responses to fluctuating thermal regimes, we collected temperature data from Yosemite toad breeding pools and cultured *Bd* under fluctuating thermal conditions that simulated pool temperatures (Fig. 1). To assess *Bd* responses to thermal fluctuation, we compared multiple reproductive life history traits of a single *Bd* isolate grown at fluctuating or constant temperatures. Our constant temperature treatments span the *Bd* thermal range and represent the daily minimum, daily mean, and daily maximum of the fluctuating temperature profile (Fig. 1b). We quantified *Bd* growth over time using measurements of culture optical density, motile zoospore counts, culture fecundity (ratio of motile zoospores to optical density), and zoosporangia viability assays. We hypothesized that fluctuating temperatures would reduce *Bd* growth as compared to *Bd* grown at the constant daily mean temperature of 17.5 °C. We predicted that exposure to daily temperature fluctuations would reduce *Bd* growth rate, fecundity, zoosporangia viability, zoospore production, and time to peak zoospore release as compared to *Bd* grown at 17.5 °C.

## Results

### Optical density growth measurements

**Fig. 2:**
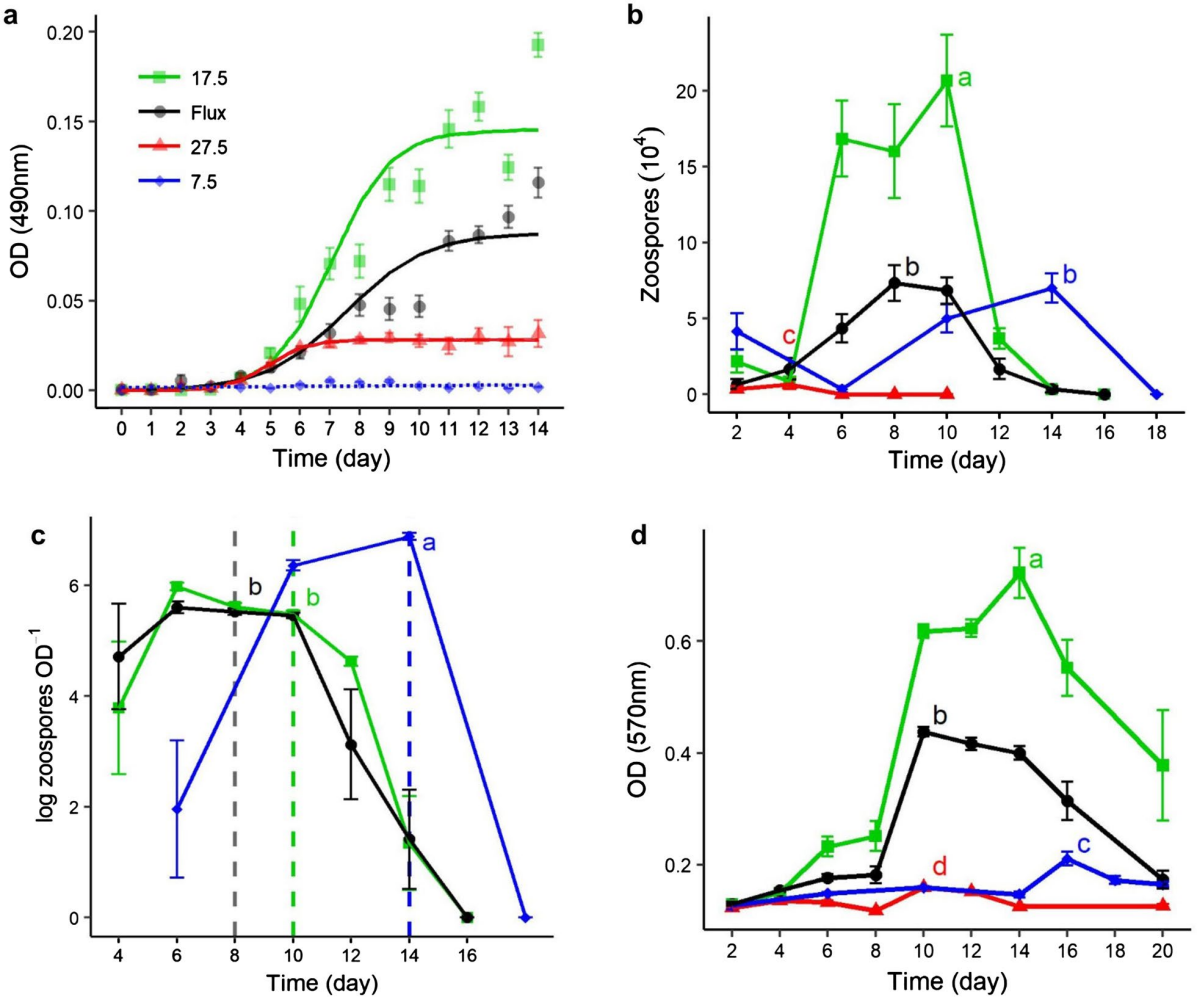
Daily fluctuating temperature reduces *Bd* growth, zoospore production, and zoosporangia viability but not culture fecundity compared to *Bd* grown at an optimal temperature. **a** Daily optical density measurements of *Bd* cultures suggest differences in maximum growth and logistic growth rate among cultures incubated at 17.5 °C (green), 27.5 °C (red), and fluctuating temperatures (black). OD readings of cultures grown at 7.5 °C (blue) did not follow a pattern of logistic growth. Points represent means (*n *= 16 treatment^−1^ day^−1^). **b** Zoospore production differed among *Bd* cultures grown at constant (7.5 °C, 17.5 °C, or 27.5 °C) and fluctuating temperatures. Cultures grown at 17.5 °C had the highest maximum zoospore production (ANOVA, *P *< 0.001). Cultures grown at 7.5 °C had similar maximum zoospore production as cultures grown under fluctuating temperatures (Tukey HSD, *P *= 0.99) but reached peak production later. Cultures grown at 27.5 °C did not produce zoospores. Points represent mean counts of motile zoospores (*n *= 6 treatment^−1^ day^−1^). **c** Fluctuating and optimal temperature cultures did not differ in fecundity. Culture fecundity was measured as the ratio of motile zoospores to total culture growth (OD) day^−1^. Despite reduced total growth (OD), cultures grown at 7.5 °C had higher fecundity than cultures grown at 17.5 °C or fluctuating temperatures (ANOVA, *P *< 0.001). Cultures grown at 27.5 °C did not produce zoospores. Dashed vertical lines correspond to time of peak zoospore production in fluctuating (day 8), 17.5 °C (day 10), and 7.5 °C (day 14) cultures. Points represent means (*n *= 6 treatment^−1^ day^−1^). **d** Zoosporangia viability differed among *Bd* cultures grown at fluctuating and constant temperatures. Cultures grown under fluctuating temperatures had lower zoosporangia viability than cultures grown at 17.5 °C but higher viability than cultures grown at 7.5 °C or at 27.5 °C (Kruskal–Wallis, *P *< 0.001). Points represent means (*n *= 8 treatment^−1^ day^−1^). In all panels, error bars represent standard error and letters indicate significant differences

We quantified total culture growth over time by measuring culture optical density (OD_490_; [51, 59, 60]), and we compared the effects of temperature treatments on *Bd* growth by fitting three-parameter logistic growth curves to OD_490_ measurements using a nonlinear mixed effects modeling approach [49]. Total culture growth differed among temperature treatments (Fig. 2a). Temperature altered *Bd* culture carrying capacity during stationary phase (asymptote; F = 201.8, *P *< 0.001), time to maximum growth rate (inflection point; F = 28.7, *P* < 0.001), and growth curve scale (scale; F = 17.1, *P* < 0.001) in 17.5 °C, 27.5 °C, and fluctuating temperature cultures (Fig. 2a). The three-parameter logistic growth model suggests that 17.5 °C cultures had the highest maximum growth, reached stationary phase after 14 days, and achieved the fastest growth rate during the exponential growth phase of all temperature treatments (Table 1). Compared to cultures grown at 17.5 °C, *Bd* grown at fluctuating temperatures had lower maximum growth and a lower maximum growth rate (Table 1). Cultures grown at 27.5 °C had lower growth rate and lower carrying capacity than optimal and fluctuating temperature cultures but reached maximum growth faster. Linear models of OD_490_ readings for 7.5 °C cultures suggested little growth over time (F = 3.15, df = 13, *P *= 0.10).

**Table 1:** Parameter estimates for logistic growth models of *Bd* grown at constant (17.5 °C and 27.5 °C) and fluctuating temperatures (7.5–27.5 °C, “Flux”)

Temp	Max. growth rate	Asymptote ± SE	p-value*	Inflection point ± SE	p-value*	Scale ± SE	p-value*
17.5 °C	0.037	0.145 ± 0.004	< 0.001	7.08 ± 0.19	< 0.001	0.97 ± 0.07	< 0.001
Flux	0.016	0.088 ± 0.006	< 0.001	7.55 ± 0.33	0.162	1.33 ± 0.15	< 0.001
27.5 °C	0.011	0.024 ± 0.005	< 0.001	4.91 ± 0.30	< 0.001	0.63 ± 0.19	0.003

* p-values reflect confidence in the model fit of each parameter

### Zoospore production

We counted motile zoospores from randomly selected wells over time to quantify zoospore production [70]. *Bd* cultures grown at 7.5 °C, 17.5 °C, 27.5 °C, or a daily fluctuating temperature profile differed in time to and productivity at peak zoospore release (Fig. 2b; ANOVA, df = 3, F = 42.2, *P *< 0.001). Between-group comparisons suggest that peak zoospore production in cultures grown at 17.5 °C was higher than in cultures grown at any other temperature profile (Tukey HSD, *P *< 0.001 for all pairwise comparisons with 17.5 °C). Peak zoospore release occurred over the same period from days 6 to 10 in fluctuating temperature and 17.5 °C cultures, but the mean maximum zoospore production was lower in fluctuating cultures (7 ± 1 × 10^4^ zoospores mL^−1^) than in 17.5 °C cultures (21 ± 3 × 10^4^ zoospores mL^−1^; Tukey HSD, *P *< 0.001). Cultures grown at 7.5 °C took the longest to reach peak zoospore production, with a mean maximum of 7 ± 1 × 10^4^ zoospores mL^−1^ on day 14. Although there was a 4–6-day difference in timing to zoospore peak, there was no difference in maximum zoospore production between *Bd* cultures grown at 7.5 °C and at fluctuating temperatures (Tukey HSD, *P *= 0.99). We did not observe new zoospore production in cultures grown at 27.5 °C.

### Culture fecundity

We measured culture fecundity as the ratio of motile zoospores to total culture growth per day. We found that fecundity differed among temperature treatments (ANOVA, F = 164.2, df = 2, *P *< 0.001), with cultures grown at 7.5 °C having higher fecundity than cultures grown at 17.5 °C or fluctuating temperatures (Fig. 2c). At peak zoospore production, cultures grown at 7.5 °C were on average 26% (15–43%) more productive per unit culture growth (OD_490_) than cultures grown at 17.5 °C (Tukey’s HSD, *P *< 0.001), and on average 23% (14–39%) more productive than cultures grown at fluctuating temperatures (Tukey’s HSD, *P* < 0.001). There was no significant difference in fecundity at peak zoospore production between cultures grown at 17.5 °C and fluctuating temperatures (Tukey’s HSD, *P *= 0.87). Cultures grown at 27.5 °C did not produce zoospores and therefore had a fecundity of zero.

### Zoosporangia viability

We used an MTT assay, a colorimetric test of cell metabolic activity, to quantify differences in culture viability over time among temperature treatments [33]. The MTT assay differentiates between living and dead cells and captures the decay phase of culture growth, providing additional growth metrics that optical density measurements cannot detect [33]. Temperature altered culture metabolic activity, with differences in maximum metabolic activity among all temperature treatments (Fig. 2d; Kruskal–Wallis, χ^2^ = 28.75, df = 3, *P *< 0.001; Conover–Iman, *P* < 0.001 for all pairwise temperature comparisons). Metabolic activity was highest in cultures grown at 17.5 °C (Conover–Iman, *P *< 0.001), with metabolic activity increasing rapidly on day 10 and peaking on day 14 (OD_570_ = 0.612 ± 0.045). Cultures grown at fluctuating temperatures also showed a rapid increase in metabolic activity, with a peak on day 10 (OD_570_ = 0.323 ± 0.009) that lasted through day 14, but fluctuating temperature cultures never surpassed optimal temperature cultures in metabolic activity (Conover–Iman, *P *< 0.001). Peak metabolic activity was lower in cultures grown at low temperatures (OD_570_ = 0.087 ± 0.012) than in cultures grown at 17.5 °C or at fluctuating temperatures and reached maximum metabolic activity on day 16 long after 17.5 °C and fluctuating temperature cultures. Although zoosporangia grown at 27.5 °C failed to produce zoospores, zoosporangia remained metabolically active at 27.5 °C for 14 d and reached peak metabolic activity (OD_570_ = 0.045 ± 0.001) on day 10.

## Discussion

Temperature fluctuations can alter the thermal performance of a pathogen and thereby the host–pathogen interactions in an infectious disease system [9, 30, 45, 55]. We examined the effect of an ecologically relevant daily temperature fluctuation on *Bd* reproductive life history traits. We found that temperatures that simulated those of breeding pools occupied by a susceptible host species reduced *Bd* growth and reproduction. Cultures grown in daily fluctuating thermal conditions had decreased total growth, growth rate, viability, and zoospore production compared to *Bd* grown at the constant daily mean temperature of 17.5 °C. Since increases in *Bd* load frequently determine the development of chytridiomycosis and resulting mortality [72, 74], reductions in *Bd* growth and zoospore production under daily fluctuating temperatures may slow disease progression in wild amphibian hosts [23].

Decreased *Bd* growth and zoospore production under fluctuating temperatures could be attributed to several factors. The time interval that *Bd* is exposed to temperatures above its thermal optimum in a diurnal cycle, and/or the magnitude of increase of the daily temperature maximum above the *Bd* thermal optimum may be important constraints on *Bd* life history traits in fluctuating thermal environments [23]. Temperature performance curves are asymmetric, with a gradual rise in performance from the thermal minimum to the optimal temperature and an abrupt drop in performance as temperatures rise above the thermal optimum [27, 37]. As such, exposure to temperatures above an organism’s thermal optimum may disproportionately affect its performance and fitness [3, 37]. These observations may be relevant for disease dynamics in Yosemite toad pool habitats where water temperatures frequently warm above 27.5 °C in summer months, with some daily temperature maxima recordings reaching 37 °C (Fig. 1a; [36]). Further, the daily temperature maximum, minimum, and range of Yosemite toad breeding pools vary across elevational, latitudinal, and seasonal gradients [36], which may contribute to temporal and spatial patterns of *Bd* persistence in the environment or abundance on hosts. As such, understanding subtleties in the duration or intensity of exposure to temperatures above a pathogen’s thermal optimum may be important in predicting disease in wild systems [15, 23, 46].

Alternatively, the decreased *Bd* growth and zoospore production we observed under fluctuating temperatures may be the result of exposure to a novel thermal environment [55, 71]. Exposure to variable and unpredictable temperatures can reduce performance of organisms, including pathogens [37, 46, 69]. As such, exposure to a novel thermal environment (i.e. exposure to fluctuating temperatures after culturing at constant optimal temperatures) could result in the decreases in growth and reproduction we observed. However, continued exposure to a predictable daily fluctuating temperature profile across multiple generations may result in a return to *Bd* growth and reproduction rates observed at optimal temperatures if the pathogen adapts to its new thermal environment [22]. Indeed, *Bd* may be able to respond to selective pressures from different thermal environments over time in vitro [71] and may grow faster under predictable rather than stochastic temperature fluctuations [55]. Adaptation to fluctuating thermal environments remains to be tested using experimental evolution approaches (e.g., serially passaging *Bd* under a variety of temperature profiles to track adaptive shifts) and could improve understanding of *Bd* persistence in wild systems, such as for Yosemite toads where the pattern of diurnal temperature fluctuation is largely predictable in summer months [36].

Our study provided several additional insights on pathogen trade-offs resulting from exposure to constant low and constant high temperatures. At constant low temperatures (7.5 °C), *Bd* cultures had reduced overall growth and a longer reproductive cycle but the highest fecundity of all temperature treatments. These results corroborate findings from *Bd* growth experiments at 7–10 °C [66, 71, 78] and provide strong evidence of fitness trade-offs that are induced by temperature. At constant high temperatures (27.5 °C), *Bd* cultures did not produce a subsequent generation of zoospores but remained metabolically active for 14 days. This result suggests that *Bd* may exhibit an additional temperature-induced trade-off in reproductive investment, reducing zoospore production to increase survival probability at or above its thermal maximum [56, 65]. This strategy has been observed in other fungi that show greater temperature constraints on spore production than on growth [25]. Further investigations into trade-offs for reproductive investment may have meaningful implications for disease development and transmission, where exposure to temperatures above the *Bd* thermal maximum may reduce zoospore production (and therefore pathogenicity) but may not fully eliminate *Bd* from hosts or the environment [14, 66, 73].

Quantifying the effect of fluctuating temperatures on *Bd* life history traits, decoupled from host defenses, provides important information for predicting pathogen growth, reproduction, and trade-offs in thermal conditions experienced by wild amphibian hosts [23, 71, 73, 78]. Important next steps include investigating how daily fluctuating temperatures affect host–pathogen interactions in vivo because *Bd* growth patterns in culture under dynamic thermal environments do not always match growth patterns on hosts [10, 55]. *Bd* must contend with host behavioral thermoregulation, host microbiome, and host immune responses in vivo [44, 57, 77]. These host defenses, pathogen growth, and their interaction can be affected by thermal conditions [10, 24, 52, 54, 61]. Further investigations into the role of realistic temperature fluctuations in relation to the thermal performance of both host and pathogen will dramatically advance our understanding of disease outcomes under natural conditions [11, 55].

## Conclusions

Our results underscore the importance of investigating pathogen life history traits under ecologically relevant conditions. Differences in magnitude, range, and variability of temperature fluctuations affect host and pathogen traits in multiple disease systems [1, 58]. Experiments that incorporate simulated natural temperature fluctuations, as opposed to temperature means, are likely to reveal important patterns in host and pathogen biology that may help identify potential climatic refugia or hotspots for infectious disease outbreaks [12, 15, 40, 67]. Because temperature maxima and variability are projected to increase at daily and seasonal scales [17, 47], it will be important to identify pathogen responses to larger and less predictable changes in temperature to better understand emerging infectious diseases in a changing climate.

## Methods

### Temperature profile selection

We collected temperature data from Yosemite toad breeding pools in ten different sites that are representative of the diversity of toad habitat across Yosemite National Park, CA. We placed one iButton (Thermochron) in each ephemeral pool at a depth that Yosemite toad tadpoles were observed to be most abundant ($\bar{x}$ = 5 cm). This stratum of observed high abundance and activity was generally at the peripheral margin of each pool with highest sun exposure and matched the narrow preference for pool microhabitat depths found in other Yosemite toad tadpole studies (4–5 cm; [31]). We recorded pool temperatures at egg deposition, which varied across sites from 07-Jun-2016 to 26-Jun-2016. Temperatures were recorded every 2 h until collection ceased between 18-Jul-2016 and 07-Aug-2016. We selected a mean fluctuating temperature profile from a single pool that diurnally oscillated between 7.5 °C and 27.5 °C, simulating thermal conditions experienced by Yosemite toads in the field (Fig. 1a). In addition, we grew *Bd* at constant temperatures of 27.5 °C, 7.5 °C, and 17.5 °C, which represent the daily high, low, and mean temperatures of the fluctuating profile respectively (Fig. 1b).

### Isolate selection, culturing, and plate set-up

We selected Sierra Nevada isolate MYLF-16343, collected from mountain yellow-legged frog (*Rana muscosa*) skin and known to cause lethal chytridiomycosis in Yosemite toads [32]. Immediately following isolation, we cryo-archived MYLF-16343 [8]. Prior to our experiment, we revived the isolate and passaged it using standard protocols [8]. When we observed peak zoospore release, we filtered cultures to remove zoosporangia, counted zoospores using a hemocytometer, and diluted the zoospore filtrate to a concentration of 46 × 10^4^ zoospores mL^−1^ [70]. We inoculated zoospores into 12 sterile, flat-bottom 96-well plates. In experimental wells, we pipetted 50 μL of zoospore filtrate and 50 μL TGhL for a final concentration of 23 × 10^4^ zoospores mL^−1^ well^−1^. In negative control wells, we pipetted 50 μL of heat-killed standardized zoospore filtrate (10-min incubation at 40 °C) and 50 μL TGhL. We randomly assigned three plates to either 7.5 °C, 17.5 °C, 27.5 °C, or fluctuating temperature (diurnal fluctuation between 7.5 and 27.5 °C, Fig. 1b) incubators (Conviron).

### Quantification of *Bd* growth

We used four methods to quantify *Bd* growth over time: (1) optical density (OD), (2) motile zoospore counts, (3) culture fecundity, and (4) zoosporangia viability assays. We measured OD of individual wells daily at 490 nm (Biotek ELx800 Absorbance Reader) to capture *Bd* logistic growth over time [51, 59, 60]. We counted motile zoospores from randomly selected wells (*n *= 6 treatment^−1^ day^−1^) using a hemocytometer to quantify zoospore production [70]. Due to the differences in life cycle times for *Bd* cultures incubating at different temperatures, we counted zoospores from plates incubated at 17.5 °C, 27.5 °C, and fluctuating temperatures every other day and from plates at 7.5 °C once every four days. We used the ratio of zoospores to culture OD per well as a metric for culture fecundity (*n *= 6 treatment^−1^ day^−1^). We used an MTT assay to measure zoosporangia metabolic activity and viability (*n *= 8 treatment^−1^ day^−1^; [33]). The MTT assay is a colorimetric test of cell viability in which the yellow tetrazolium salt MTT (3-(4,5-dimethylthiazol-2-yl)-2,5-diphenyltetrazolium bromide) is reduced to purple MTT-formazan crystals in metabolically active cells [34, 42]. This color change can be quantified by solubilizing the crystals and measuring culture absorbance at a sensitive wavelength (570 nm, [2]). We destructively sampled wells used for zoospore counts and zoosporangia viability assays.

### Statistical analysis

We conducted all analyses using R v3.4.3 [53]. Unless otherwise noted, summary statistics in figures and text represent mean ± standard error (SE). We fit three-parameter logistic curves to OD measurements of culture growth using nonlinear mixed effects models with the ‘nlme’ package v3.1.131.1 in R [50]. Three-parameter logistic models can be used to predict the culture stationary phase (i.e., asymptote), the time at which cultures are in exponential growth phase halfway to stationary phase (i.e., inflection point), and a scale parameter that determines the steepness of the growth curve. We included temperature treatment fixed effects and well random effects on all parameters. We addressed within-group error heteroscedasticity by adding an identity variance structure to the model [49]. We corrected for initial zoospore inoculation and media color by subtracting OD values of heat-killed controls from OD values of wells containing *Bd* and ran models using wells that were not used for zoospore counts or MTT assays (*n *= 16). We used analysis of variance (ANOVA) and likelihood ratio tests for model selection and tested for the joint significance of the temperature fixed effect with an *F*-test. For OD measurements of cultures grown at a constant temperature of 7.5 °C, *Bd* growth was slow and minimal such that nonlinear mixed effects models did not converge. We fit a linear model to OD measurements from these low temperature cultures.

To compare differences in peak zoospore production among temperature treatments, we used ANOVA and Tukey’s HSD post hoc tests. We used a square root transformation for the zoospore counts to achieve homogeneity of variance among temperature treatments. To compare differences in zoosporangia viability at peak culture metabolic activity, we used a Kruskal–Wallis test. We looked at pairwise comparisons between temperature treatments using a Conover–Iman post hoc test with a Bonferroni correction. To evaluate fecundity, we used a ratio of zoospores produced to mean culture OD day^−1^. We log-transformed this fecundity metric and added a correction factor of 1 to allow for log-transformation of values from wells with zero fecundity (no zoospores produced). We compared differences in culture fecundity at peak zoospore production using ANOVA and Tukey’s HSD post hoc tests and back-transformed percent differences in the reported fecundity results.

## Data Availability

The datasets used and analyzed during the current study are available from the corresponding author on reasonable request.
